# Induction of endothelial cell apoptosis by the antivascular agent 5,6-dimethylxanthenone-4-acetic acid

**DOI:** 10.1038/sj.bjc.6600368

**Published:** 2002-06-17

**Authors:** L-M Ching, Z Cao, C Kieda, S Zwain, M B Jameson, B C Baguley

**Affiliations:** Auckland Cancer Society Research Centre, University of Auckland School of Medicine, Private Bag 92019, Auckland, New Zealand; Centre de Biophysique Moleculaire, CNRS UPR4301, Orléans, France

**Keywords:** DMXAA, apoptosis, antivascular, Colon 38 tumour, endothelial

## Abstract

5,6-Dimethylxanthenone-4-acetic acid, synthesised in this laboratory, reduces tumour blood flow, both in mice and in patients on Phase I trial. We used TUNEL (TdT-mediated dUTP nick end labelling) assays to investigate whether apoptosis induction was involved in its antivascular effect. 5,6-Dimethylxanthenone-4-acetic acid induced dose-dependent apoptosis *in vitro* in HECPP murine endothelial cells in the absence of up-regulation of mRNA for tumour necrosis factor. Selective apoptosis of endothelial cells was detected *in vivo* in sections of Colon 38 tumours in mice within 30 min of administration of 5,6-Dimethylxanthenone-4-acetic acid (25 mg kg^−1^). TUNEL staining intensified with time and after 3 h, necrosis of adjacent tumour tissue was observed. Apoptosis of central vessels in splenic white pulp was also detected in tumour-bearing mice but not in mice without tumours. Apoptosis was not observed in liver tissue. No apoptosis was observed with the inactive analogue 8-methylxanthenone-4-acetic acid. Positive TUNEL staining of tumour vascular endothelium was evident in one patient in a Phase I clinical trial, from a breast tumour biopsy taken 3 and 24 h after infusion of 5,6-Dimethylxanthenone-4-acetic acid (3.1 mg m^−2^). Tumour necrosis and the production of tumour tumour necrosis factor were not observed. No apoptotic staining was seen in tumour biopsies taken from two other patients (doses of 3.7 and 4.9 mg m^−2^). We conclude that 5,6-Dimethylxanthenone-4-acetic acid can induce vascular endothelial cell apoptosis in some murine and human tumours. The action is rapid and appears to be independent of tumour necrosis factor induction.

*British Journal of Cancer* (2002) **86**, 1937–1942. doi:10.1038/sj.bjc.6600368 www.bjcancer.com

© 2002 Cancer Research UK

## 

DMXAA (5,6-dimethylxanthenone-4-acetic acid), a new anticancer agent synthesised in this laboratory (Rewcastle *et al*, 1991), has recently completed Phase I clinical trial as an antivascular agent. In preclinical studies, DMXAA was particularly effective against transplantable murine tumours with an established vasculature (Rewcastle *et al*, 1991), where it caused cessation of tumour blood flow, vascular collapse and tumour necrosis (Zwi *et al*, 1994a; Lash *et al*, 1998). DMXAA increased tumour necrosis factor (TNF) concentrations in plasma of both tumour bearing and non-tumour bearing mice (Philpott *et al*, 1995). It induced TNF synthesis in both host and tumour cells of Colon 38 tumours (Joseph *et al*, 1999) and also induced significant amounts of TNF in murine Colon 38 tumours implanted in TNF knockout mice (Ching *et al*, 1999). DMXAA also induced TNF synthesis in host cells, and in some cases tumour cells, of a series of human tumour xenografts (Joseph *et al*, 1999). Co-administration of anti-TNF antibody together with DMXAA partially reversed the blood flow inhibition and antitumour action of DMXAA, suggesting that TNF plays a role in its antivascular effects (Browne *et al*, 1998).

In contrast to the large increases in plasma TNF observed in mice treated with DMXAA, plasma TNF levels in patients treated in a Cancer Research UK phase I trial were not elevated (Jameson *et al*, 2000). However, dynamic contrast-enhanced magnetic resonance imaging suggested that tumour blood flow was inhibited in most patients receiving DMXAA at doses of 500 mg m^−2^ or higher (Rustin *et al*, 1998). These studies suggested that at least some of the antivascular effects of DMXAA might be independent of plasma TNF production and result from a direct effect on tumour vasculature. In this report, we have searched for direct evidence of such an effect, using both *in vitro* and *in vivo* murine models and tumour biopsies from three patients treated with DMXAA in a Phase I trial.

## MATERIALS AND METHODS

### Materials

DMXAA was synthesised in this laboratory (Rewcastle *et al*, 1991) and dissolved in 5% sodium bicarbonate for intraperitoneal injection into mice (25 mg kg^−1^) in a volume of 0.01 ml g^−1^ body weight. DMXAA was dissolved directly in culture medium for *in vitro* experiments. In the clinical phase trial conducted in Auckland, New Zealand, DMXAA was administered as a 20 min intravenous infusion on a 3-weekly schedule, using a pre-formulated solution of 20 mg ml^−1^ in 0.1 M phosphate buffer at pH 7.7 (Jameson *et al*, 2000).

### Mouse studies

Athymic or normal C57Bl/6 mice were from the Animal Laboratories, University of Auckland School of Medicine. All mice were maintained under constant temperature and humidity according to institutional ethical guidelines and used between 8–12 weeks of age. All animal experiments have been carried out with ethical committee approval. The ethical guidelines that were followed meet the standards required by the UKCCCR guidelines (Workman *et al*, 1998). Colon 38 tumour fragments (1 mm^3^) were implanted subcutaneously in the left flank of anaesthetised (sodium pentobarbitone, 86 mg kg^−1^) normal C57Bl/6 mice. Tumours were used when they had reached approximately 6 mm in diameter, generally 9–10 days after implantation. Three mice were used for each time point and treatment, and 2–4 cryosections per organ per mouse were immunostained for apoptosis using TUNEL.

### Clinical studies

Three patients with superficial tumours (two with chest wall recurrence of breast adenocarcinoma and one with subcutaneous ovarian adenocarcinoma tumour nodules) consented to biopsies. These samples were taken prior to infusion of DMXAA then at various intervals afterwards and were embedded (OCT compound, a mixture of water-soluble glycols and resins, Tissue-Tek®, Sakura Finetech, Torrance, CA, USA) and snap-frozen. Representative cryosections were immunostained for apoptosis using TUNEL.

### *In vitro* studies

The HECPP murine endothelial cell line derived from endothelial cells isolated from murine Peyer's patches (Bizouarne *et al*, 1993) was maintained in M199 media (Gibco BRL) with antibiotics and 10% foetal calf serum at 37°C under humidified atmosphere of 5% CO_2_. Sub-confluent cultures (that had been passaged the previous day) were incubated with DMXAA for the appropriate time. The supernatant containing non-adherent cells was removed and centrifuged at 300×**g** for 10 min. Adherent cells were lifted from the dishes by trypsinisation and collected similarly. Combined adherent and supernatant cells, resuspended in culture medium (0.4 ml), were used to prepared cytospin preparations that were assayed for apoptosis.

### Apoptosis assay

Apoptosis was determined using the TUNEL assay for identification of double-stranded DNA breaks using an *In Situ* Cell Death Detection Kit (Boehringer Mannheim) according to manufacturer's instructions. Tissue and tumour cryosections or cytospots of cells on poly-L-lysine-treated slides were fixed in 4% paraformaldehyde for 30 min at room temperature, washed with PBS (phosphate buffered saline) and then treated with permeabilisation solution (0.1% Triton X-100 in 0.1% sodium citrate) for 2 min on ice. Strand breaks were labelled with fluoresceinated dUTP and visualised following reaction with phosphatase-conjugated antibody to fluorescein and Vector® Black alkaline phosphatase substrate solution (Vector Laboratories, Burlingame, CA, USA). All slides were counter stained using methyl green.

Cryosections of Colon 38 tumour tissues were also stained for endothelial cells to differentiate between apoptosis of the endothelium and apoptosis of tumour cells. Sections were first processed for apoptosis as described and then immunostained for endothelial cells using a rat anti-mouse CD-31 antibody (MEC 13.3, a generous gift from Dr A Vecchi, Instituto di Rierche Farmacologie Mario Negri, Via Eritrea, Milan, Italy) (Vecchi *et al*, 1994), followed by incubation with biotinylated anti-rat IgG secondary antibody (Vector Laboratories, Burlingame, CA, USA) and the avidin-biotin complex (Vectastain® ABC-AP Kit). Immunoglobulin complexes were visualised using Vector® Red alkaline phosphatase substrate solution.

### Northern blot analysis

Total cellular RNA was extracted using RNAzol (Gibco, BRL) according to manufacturer's instructions. RNA (10 μg) was denatured and electrophoresed in 1% agarose-formaldehyde gels as previously described (Ching *et al*, 1994). RNA was then transferred by capillary action onto nylon membrane (Hybond-N^+^, Amersham). The membranes were UV-crosslinked (120 mJoule, UV-Stratalinker, Stratagene, San Diego, CA, USA) and baked (80°C for 30 min). Each membrane was prehybridised (2 h, 42°C) in 7 ml hybridisation mix containing 50% formamide, 0.075 M sodium chloride, 0.05 M sodium dihydrogen phosphate, 5 mM EDTA, 0.001% polyvinyl pyrrolidone, 0.001% bovine serum albumin, 0.001% Ficoll, 0.01 mg ml^−1^ herring sperm DNA, and 0.5% SDS (sodium dodecyl sulphate). The cDNA to the cytokine gene of interest was labelled with α^32^P-dCTP (Amersham) using a random priming kit (RTS Radprime DNA labelling system, Gibco BRL). Excess radioactivity was removed by elution through a G-50 Sephadex column and labelled probe (10^6^ c.p.m. ml^−1^ hybridisation mix) was then added to the membrane and hybridised for 36 h at 42°C. The blots were washed twice in 2×SSC (standard saline citrate) with 0.1% SDS for 10 min at 42°C, and finally in 0.2×SSC with 0.1% SDS for 10 min at 65°C. Blots were exposed to X-ray film for 1–3 days at −70°C. After hybridisation with one probe, membranes were stripped (two washes in 300 ml 0.1×SSC with 1% SDS for 15 min at 80°C) and re-hybridised with another probe. The signal intensity was quantitated by laser densitometric scanning. Loading of lanes was determined from the intensity of bands hybridised with the probe for glyceraldehyde-3-phosphate dehydrogenase (GADPH).

## RESULTS

### Induction of endothelial cell apoptosis in murine tissues following DMXAA

Sections of Colon 38 tumours, removed at different times from C57Bl/6 mice administered DMXAA at its maximum tolerated dose (25 mg kg^−1^), were double stained for apoptosis and for CD31expression (Table 1

**Table 1:**
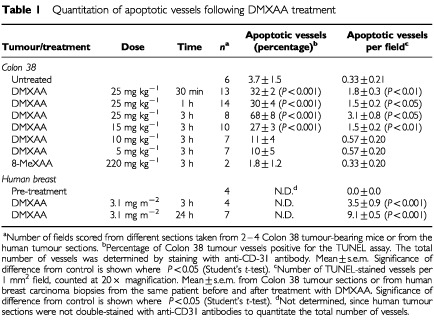
Quantitation of apoptotic vessels following DMXAA treatment

). As early as 30 min after treatment, faint apoptotic staining above background staining of untreated controls (Figure 1A

**Figure 1:**
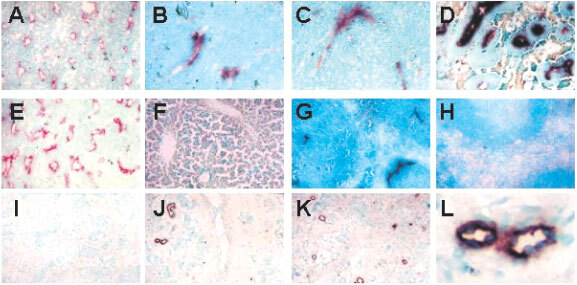
Induction of endothelial cell apoptosis by DMXAA. (**A**–**E**) Representative cryosections of Colon 38 tumours (100× magnification) were immunostained for apoptosis using TUNEL (black) and endothelial cells with antibodies to CD-31 (red): (**A**) untreated, (**B**) 30 min after DMXAA (25 mg kg^−1^), (**C**) 1 h after DMXAA, (**D**) 3 h after DMXAA, (**E**) 3 h after 8-MeXAA (220 mg kg^−1^). (**F**–**H**) Representative cryosections (100× magnification) immunostained for apoptosis using TUNEL (black): (**F**) liver tissue from Colon 38 tumour-bearing mice 3 h after DMXAA (25 mg kg^−1^), (**G**) spleen tissue from Colon 38-bearing mice 3 h after DMXAA (25 mg kg^−1^), and (**H**) spleen tissue from normal mice 3 h after DMXAA (25 mg kg^−1^). (**I**–**L**) Representative cryosections (100× magnification) from a human breast carcinoma immunostained for apoptosis using TUNEL (black): (**I**) before treatment, (**J**) 3 h after DMXAA infusion (3.1 mg m^−2^), (**K**) 24 h after DMXAA. (**L**) Section at higher magnification (500×) of two vessels 3 h after DMXAA infusion.

) was detected and found to be associated with the endothelial cells in the Colon 38 tumour (Figure 1B). The staining intensity, as well as the number of apoptotic vessels, increased progressively with time after treatment (Table 1). Up to 3 h after treatment, apoptosis was associated only with the tumour vascular endothelium, with no apoptosis of tumour cells (Figure 1B,C). Colon 38 sections taken 3 h after DMXAA administration contained intensely stained apoptotic vessels, and at this time, large areas of necrosis of the tumour were observed (Figure 1D). Following the maximal tolerated dose of DMXAA, 69% of the vessels in the tumour were apoptotic, while after a dose of 15 mg kg^−1^, 27% were apoptotic. Significant apoptosis of vessels was not observed following DMXAA doses of 5 or 10 mg kg^−1^ (Table 1). Colon 38 tumour sections removed from mice 3 h after treatment with an inactive analogue, 8-MeXAA (8-methylxanthenone-4-acetic acid) at its maximal tolerated dose (220 mg kg^−1^) showed no apoptotic vessels or tumour necrosis (Figure 1E; Table 1). No staining was observed with liver sections from either normal or tumour-bearing mice, before or 3 h after DMXAA (25 mg kg^−1^) (Figure 1F). Strong staining for apoptosis was observed in the central vessel in the white pulp regions of spleens from all tumour-bearing mice 3 h after DMXAA (Figure 1G), and was not present without treatment. Apoptosis in the splenic vasculature was tumour-dependent, since apoptotic cells were not observed in spleens from normal mice following DMXAA administration (Figure 1H).

### Endothelial cell apoptosis in human tumours following DMXAA

Tumour biopsies from one patient with breast adenocarcinoma prior to DMXAA infusion (3.1 mg m^−2^) did not stain with the TUNEL assay (Figure 1I). However apoptotic staining in vessels was seen in the biopsy taken 3 h post-infusion (Figure 1J) and the number of apoptotic vessels increased three-fold in the biopsy taken after 24 h (Figure 1K; Table 1). In contrast with the murine 3 h samples, haemorrhagic necrosis was not observed. In another two patients administered DMXAA at 3.7 and 4.9 mg m^−2^ no staining for apoptosis was seen in samples taken prior to drug infusion nor in breast carcinoma samples taken at 3 and 24 h or ovarian carcinoma samples taken at 4.5 h.

### DMXAA induces HECPP endothelial cell apoptosis in culture

**Figure 2:**
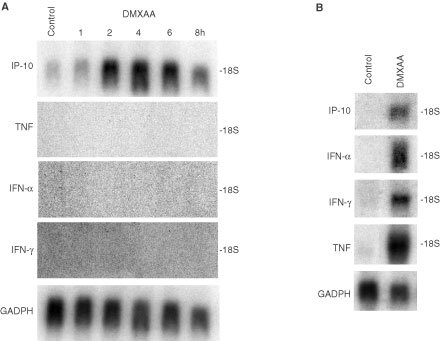
Northern blots for mRNA expression. (**A**) mRNA for IP-10, TNF, IFN-α, IFN-γ in HECPP cells detected 2 h following *in vitro* treatment with DMXAA (400 μg ml^−1^). Loading of each lane shown by GADPH mRNA levels. Untreated controls (lane C). (**B**) mRNA for IP-10, TNF, IFN-α, IFN-γ in murine splenocytes detected 2 h following *in vivo* treatment with DMXAA (25 mg kg^−1^).

**Figure 3:**
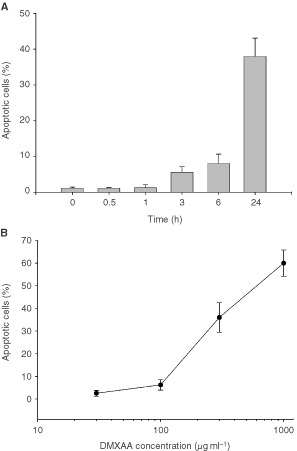
Induction of apoptosis by DMXAA in cultured HECPP endothelial cells. (**A**) Percentage of HECPP cells stained using TUNEL assay for apoptosis after various incubation times with DMXAA (400 μg ml^−1^). (**B**) Percentage TUNEL-staining HECPP cells 24 h after exposure to different DMXAA concentrations. Mean±s.e.m. of at least three determinations, obtained by counting 500 cells.

We used the murine endothelial HECPP cell line (Bizouarne *et al*, 1993) to determine whether DMXAA could induce apoptosis *in vitro* in the absence of TNF induction. As seen from Northern blot analyses, DMXAA does not induce mRNA for TNF or interferons in the HECPP cells (Figure 2A

). Of the cytokine genes that are up-regulated in mice following DMXAA treatment (Figure 2B), only mRNA for IP-10 was up-regulated following 2 h incubation with DMXAA at 400 μg ml^−1^ in the HECPP cells (Figure 2A). HECPP cells treated with DMXAA were processed using TUNEL assays and the percentages of apoptotic cells were determined by counting at least 500 cells. Following exposure to DMXAA at a concentration (400 μg ml^−1^) that is achievable *in vivo* after administration of an effective antitumour dose (McKeage *et al*, 1991), apoptotic cells were seen after 6 h incubation, and the numbers increased with prolonged exposure (Figure 3A

). Apoptotic cell numbers at 24 h increased linearly with increasing dose of DMXAA above 100 μg ml^−1^. The DMXAA concentration that induced 50% apoptosis after incubation for 24 h was 500 μg ml^−1^ (Figure 3B).

## DISCUSSION

These results are the first to demonstrate the selective induction of endothelial cell apoptosis in both a murine tumour (Figure 1B–D) and a human tumour (Figure 1J–L) following treatment with DMXAA. Loss of tumour vascular endothelial cells by apoptosis would be expected to increase the permeability of the vascular endothelium, providing a potential mechanism for reduction in tumour blood flow (Baguley, 2001) as demonstrated in both murine models (Zwi *et al*, 1994a,b; Lash *et al*, 1998) and in clinical studies (Rustin *et al*, 1998). The results also provide a possible mechanism for the DMXAA-induced extravasation of erythrocytes in murine tumours (Zwi *et al*, 1994b). Although a number of studies have implicated TNF in the antitumour response of DMXAA (Browne *et al*, 1998; Cao *et al*, 1999), three observations in this study support a TNF-independent action of DMXAA on the vascular endothelium. Firstly, apoptosis of endothelial cells was observed in Colon 38 tumour sections within 30 min after administration, well before the time at which TNF is detectable in plasma or tumour tissue (Joseph *et al*, 1999). Secondly, in the patient where apoptosis was induced in tumour vessels following treatment with DMXAA, no TNF induction was detectable in tumour tissue or plasma (Jameson *et al*, 2000). Thirdly, apoptosis was induced with increasing concentrations of DMXAA in HECPP cells in culture (Figure 3) when the cells did not produce TNF (Figure 2A). Of all the cytokine genes that are known to be induced *in vivo* by DMXAA in murine tissues (Cao *et al*, 2001), only mRNA for IP-10 was up-regulated in cultures of HECPP cells (Figure 2A). IP-10 is known for its chemotactic (Luster *et al*, 1985; Taub *et al*, 1993) and anti-angiogenic activities (Angiolillo *et al*, 1995) but has no reported apoptotic activity. Our results here suggest that DMXAA may have a direct apoptotic effect on endothelial cells.

The induction of endothelial apoptosis in tumour tissue by DMXAA is not completely tumour-selective. While no apoptosis was observed in sections from liver of normal or tumour-bearing mice (Figure 1E), apoptotic cells were detected in the vasculature of spleens from tumour-bearing animals (Figure 1G). Since such cells were not observed in spleens of normal mice (Figure 1H), the tumour appears to sensitise endothelial cells in spleen, but not liver, to apoptosis induction by DMXAA. A possible reason for this is that factors released from tumours activate splenocytes, which then react to DMXAA by releasing TNF, thus inducing endothelial cell apoptosis and reducing splenic blood flow. The results are consistent with the observation that splenic perfusion was transiently and selectively lowered in tumour-bearing mice following treatment with FAA (flavone-8-acetic acid), a drug related structurally to DMXAA (Honess and Bleehen, 1991). Previous work from our laboratory has indicated the presence of tumour-secreted factors leading to priming of tumoricidal macrophages and their responsiveness to activation by DMXAA (Ching *et al*, 1993). The procoagulant activity of human endothelial cells in culture is strongly up-regulated by conditioned growth medium from a cultured human breast adenocarcinoma cell line. The tumour conditioned medium had a greater effect on endothelial cells than did hypoxia, reduced oxygen tension, TNF, FAA or DMXAA (Watts *et al*, 1996), providing another clear example of the priming effect of tumour secreted factors.

The ‘priming’ effect may provide an explanation of the remarkably rapid onset (30 min) of apoptosis of vascular endothelial cells in the Colon 38 tumour. It is not known whether vascular endothelial apoptosis could occur as rapidly in human tumours, since the earliest time point following DMXAA that we were able to examine was 3 h. However, apoptosis occurred more rapidly following DMXAA administration *in vivo* than in HECPP cells in culture.

Clinical trials of DMXAA have indicated a low response rate to DMXAA (Jameson *et al*, 2000). However, good evidence has been provided for induced vascular effects, such as reduced tumour blood flow (Rustin *et al*, 1998), serotonin release (Jameson *et al*, 2000) and the endothelial apoptosis reported in this study. The clinical trial results differ from those of murine studies in that although TNF synthesis was detected in one human tumour sample (Jameson *et al*, 2000) the level was low and TNF was not detected in the sample in which endothelial cell apoptosis was observed. Widespread haemorrhagic necrosis is a prominent effect of DMXAA treatment in murine tumours (Baguley *et al*, 1989; Zwi *et al*, 1994b; Cao *et al*, 1999) but has not so far been observed in clinical trials. In the Colon 38 tumour, *in situ* TNF synthesis (Joseph *et al*, 1999) is probably responsible for sustaining the vascular effect of DMXAA and inducing haemorrhagic necrosis. Combination of DMXAA with agents that improve intratumoural TNF synthesis might constitute a rational approach in further clinical trials of DMXAA. In cultured human peripheral blood leucocytes, a second signal, such as that provided by trace amounts of endotoxin or interleukin-1, is required in order for DMXAA to induce TNF production (Philpott *et al*, 2001). Clinical studies employing DMXAA in combination with other agents that provide a second signal within tumour tissue may provide a pathway towards improved anticancer therapy.
